# The Evolution of Lipidomics during Oil Accumulation of *Plukenetia volubilis* Seeds

**DOI:** 10.3390/plants13162193

**Published:** 2024-08-08

**Authors:** Yijun Fu, Qiongjian Ou, Lixuan Ye, Huiyan You, Zhaohui Wang, Ao Yi, Jia Wang, Jun Niu

**Affiliations:** 1 Key Laboratory of Genetics and Germplasm Innovation of Tropical Special Forest Trees and Ornamental Plants-Ministry of Education, School of Tropical Agriculture and Forestry, Hainan University, Haikou 570228, China; fuyijun980614@163.com (Y.F.); oqj1219@163.com (Q.O.); xiaoyetongxue6@outlook.com (L.Y.); wybplyx666666@163.com (H.Y.); wzh08080725@163.com (Z.W.); 23220953000030@hainanu.edu.cn (A.Y.); 2 School of Information and Communication Engineering, Hainan University, Haikou 570228, China

**Keywords:** Sacha inchi, oil seeds, LC-MS/MS analysis, lipidomics

## Abstract

Sacha inchi (*Plukenetia volubilis*) is a valuable oilseed crop with a high content of polyunsaturated fatty acids (PUFAs). However, there is a lack of in-depth understanding of the lipidomics in Sacha inchi seeds (SIDs). Saturated fatty acids occupied more than half of the proportion (59.31%) in early development, while PUFAs accounted for 78.92% at maturation. The main triacylglycerols were TAG(18:3/18:3/18:3), TAG(18:2/18:2/18:3), and TAG(16:0/18:2/18:2). The corresponding species (18:3/18:3, 18:2/18:2, and 16:0/18:2) were also the main ingredients in diacylglycerol and phosphatidic acid, indicating high PUFA composition in the *sn*-1 and *sn*-2 positions of TAG. Only LPC(18:3), LPC(18:2), and LPC(16:0) were identified in SIDs, implying that those PUFAs on the *sn*-2 positions of the PC(18:3/-), PC(18:2/-), and PC(16:0/-) categories were released into the acyl-CoA pool for the Kennedy pathway. Conversely, the PC(18:1/-) and PC(18:0/-) categories might be responsible for the generation of PC-derived DAG and TAG. The lipidomics data will contribute to understanding the TAG assembly in developing SIDs, especially for PUFAs.

## 1. Introduction

Sacha inchi (*Plukenetia volubilis*), belonging to the family Euphorbiaceae, is a woody liana and is cultivated as an economic crop [1]. The oleaginous seeds of this species are the main edible organ for the indigenous people of the Amazon rainforest. Historically, Sacha inchi seeds (SIDs) can be consumed for diverse culinary uses, such as *inchi cucho*, *lechona api*, and *inchi capi* [2]. Compositional analysis of mature SIDs indicates that the first main ingredient is oil (33~58%), followed by proteins (22~30%) and other bioactive ingredients [2]. It is worth taking note that SID oil was awarded the gold medal at the ‘World Edible Oil’ contest over three consecutive years from 2004 to 2006 [1]. The main fatty acids (FAs) of SID oil were polyunsaturated FAs (PUFAs), including α-linolenic acid (C18:3) and linoleic acid (C18:2) accounting for ∼50% and ∼35% of the total oil content, respectively [3]. As the market demand for PUFAs increases, Sacha inchi has become a promising economic crop to widely apply in the production of foods, medications, cosmetics, and other fields [1,4]. The development of high-PUFA crops or oils can lead to new market opportunities and economic growth in agriculture and related industries.

In plants, the pathway of oil accumulation is mainly composed of FA biosynthesis, FA desaturation, triacylglycerol (TAG) assembly, and oil body formation [5]. In cell plastid, the generated acetyl-CoA is used as a precursor for FA biosynthesis to produce palmitic acid (C16:0) and stearic acid (C18:0). Subsequently, the monounsaturated FAs (MUFAs) and PUFAs are formed in the plastid and endoplasmic reticulum (ER) by corresponding FA desaturases (FADs), respectively [6]. In sharp contrast to the clear and single channel of FA biosynthesis and desaturation, TAG assembly is complex and varied. For example, free FAs can be assembled into TAG by the classic Kennedy pathway, the FA chain in phosphatidyl choline (PC) can be interchanged with the FA chain in diacylglycerol (DAG), or the FA chains in PC can also be used for TAG generation [5]. The resulting TAG is eventually turned into an oil body for long-term preservation in the seed [7]. Although FA compositions are clear in developing SIDs, very little is currently known about lipid changes during oil accumulation. The clarification of lipid composition may lead to an understanding of the processes of PUFA biosynthesis and assembly.

Since the concept of lipidomics was proposed in 2003, lipometabolism has attracted great attention from researchers owing to the innovation of mass spectrometry [8]. In this process, lipids are converted into ionized substances measurable by mass spectrometry, thereby enabling the identification of lipid species and their structural characteristics for a comprehensive analysis of all lipid types, their contents, and interrelations within the sample [9]. Recent lipidomics in higher plants were applied in the characterization of changes in lipid profiles [9,10,11,12]. Lipidomics information during SID oil accumulation is unavailable. The purpose of this study was to investigate the changes in lipid profiles in developing SIDs. This study provides a basis for improving our understanding of FA assembly, in particular PUFA fractions. These findings are informative for breeding programs to enhance PUFA content in crops, thereby improving the nutritional quality of food products. 

## 2. Results and Discussion

### 2.1. Oil Content and FA Compositions

**Figure 1:**
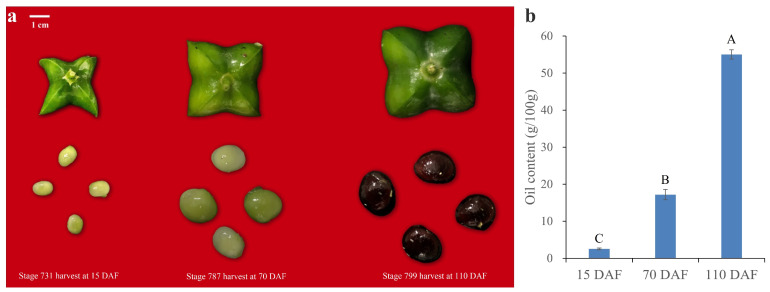
The developmental stages of *P. volubilis* fruits. (**a**) The fruits and seeds of *P. volubilis* at different developmental stages. The scale bar represents 1 cm. (**b**) The oil content of *P. volubilis* seeds during development. The oil was obtained by Soxhlet extraction. Values are represented as means ± standard deviations. Different letters above the groups represent significant differences, and shared letters represent no significant differences (*p <* 0.01).

Oilseed crops with high yield and oil content are more attractive with increasing demand for vegetable oils. Previous investigations on *P. volubilis* have revealed a sigmoid pattern of oil accumulation throughout seed development [13,14]. Importantly, the pivotal stages of oil accumulation, namely initiation, rapid accumulation, and culmination, occurred at 15, 70, and 110 days after flowering (DAFs), respectively. According to the BBCH scale [15], the three crucial time points of fruit development corresponded to stages 731, 787, and 799, which we selected to explore the lipid compositions (Figure 1a). Our results showed that the SID oil contents displayed a gradual increase with maturity from 2.51% to 55.04% (Figure 1b). This was in line with a previous report that the oil content of mature SIDs ranges from 35 to 60% [3]. The fluctuation may be correlated with climatic conditions, cultivation methods, extraction methods, and cultivars [2,16,17].

Six major FA components of SID oil were palmitic acid (C16:0), stearic acid (C18:0), oleic acid (C18:1), linoleic acid (C18:2), and α-linolenic acid (C18:3) (Table 1). In early development, the saturated FAs of palmitic and stearic acid occupied more than half of the proportion (59.31%). As the seeds developed, PUFAs gradually became the main FA compositions, accounting for 78.92% at the termination stage of oil accumulation (Table 1). Interestingly, the percentage of PUFAs was lower than the previous report of 83.44%, while the percentage of oleic acid (10.98%) was higher than the previous report of 7.96% [13]. It is undeniable that fluctuating climatic conditions inevitably lead to variations in the FA composition of oil seeds [16,17]. Studies have reported that elevated PUFA levels in membrane lipids decrease the thermotolerance of plant cells [18]. Thus, the lower PUFA content observed in this study might be due to the higher ambient temperatures in Hainan Island compared to Kunming [13], thereby enhancing plant thermotolerance. It is well established that oleic acid, a monounsaturated fatty acid (MUFA), is the principal precursor for PUFA biosynthesis [14,17]. The inhibition of PUFA synthesis results in the accumulation of MUFAs.

### 2.2. Changes in TAG Ingredients during Oil Accumulation

**Figure 2:**
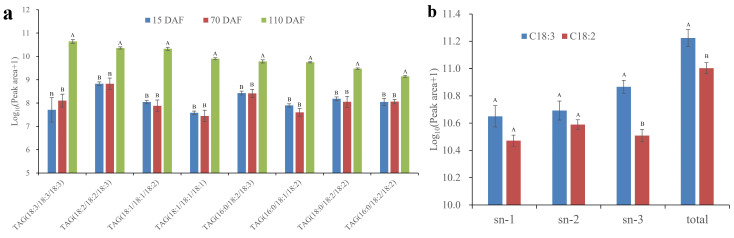
Relative content of the major TAG species during oil accumulation. (**a**) Relative contents of the major TAG species in SIDs at different developmental stages. Please refer to attached Table S1 for details. (**b**) Relative contents of C18:3 and C18:2 at different positions in mature SIDs. *sn*-1, *sn*-2, and *sn*-3, respectively, refer to different sites on the TAG. Values are represented as means ± standard deviations. Different letters above groups represent significant differences, and shared letters represent no significant differences (*p <* 0.01).

Most current research on the lipid profiles of plant seeds study the total content of different lipid classes [17,19,20], such as TAG, DAG, and PC, but detailed reports on the lipid compositions are lacking. In this study, a total of 60 TAG ingredients were identified using LC-MS/MS analysis (Supplementary Table S1). The main ingredients were TAG(18:3/18:3/18:3), TAG(18:2/18:2/18:3), TAG(18:1/18:1/18:2), TAG(18:1/18:1/18:1), TAG(16:0/18:2/18:3), and TAG(16:0/18:2/18:2), all of whose contents exhibited a notable increase at 110 DAFs (Figure 2a). After statistical calculation, the total peak area of C18:3 was higher than that of C18:2 in mature SIDs (Figure 2b). In plants, seed oil is stored mainly in TAG molecules [21]. Thus, this is not surprising given that the ratio of C18:3/C18:2 in TAG ingredients exhibited a moderate correlation with FA compositions in mature SIDs (Table 1 and Figure 2b).

It was clear that the highest content of C18:3 was in the *sn*-3 position, whereas the highest content of C18:2 was in the *sn*-2 position (Figure 2b). This result implied that the different positions of TAG might have different binding preferences for FA ligands. What was interesting about the data in Figure 2b was that, compared with *Brassica napus* [12] and *Prunus sibirica* [22], *P. volubilis* had higher C18:3 and C18:2 content in the *sn*-1 position of TAG. It seems that the *sn*-1 assembly of C18:3 and C18:2 may be important for high PUFA content in SID oil. Glycerol-3-phosphate is initially acylated by glycerol-3-phosphate acyltransferase (GPAT). Previous transcriptome profiling studies have identified five *GPAT* genes in SIDs [14]. To develop oil crops with a high PUFA content, further investigation is required into the functional divergence of these GPAT members and their correlation with PUFA assembly at the *sn*-1 position.

### 2.3. Changes in DAG Ingredients during Oil Accumulation

**Figure 3:**
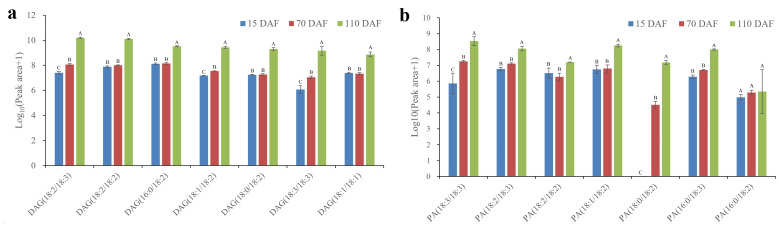
Relative contents of the major (**a**) DAG and (**b**) PA species found in SIDs during development. Please refer to attached Tables S2 and S3 for details. Values are represented as means ± standard deviations. Different letters above the groups represent significant differences, and shared letters represent no significant differences (*p <* 0.01). Abbreviations: diacylglycerol, DAG; phosphatidic acid, PA.

DAG is a lipid intermediate that is the substrate for the *sn*-3 assembly of TAG [5]. A total of 21 DAG ingredients were identified in this study (Supplementary Table S2). The main constituents were DAG(18:2/18:3), DAG(18:2/18:2), DAG(16:0/18:2), DAG(18:1/18:2), DAG(18:0/18:2), DAG(18:3/18:3), and DAG(18:1/18:1) (Figure 3a). Their content displayed a similar increasing trend during oil accumulation with the main TAG ingredients. The corresponding esterification products can also be found in TAG (Supplementary Table S1). The above results indicated that our lipidomics data were reliable. Perhaps the most surprising aspect of the data presented here is the high content of DAG(18:3/18:3) at 110 DAFs (Figure 3a), providing further evidence for the importance of PUFA assembly in the *sn*-1 position.

### 2.4. Changes in the Ingredients of Phosphatidic Acid during Oil Accumulation

In the Kennedy pathway, the first two acylations of glycerol-3-phosphate produce phosphatidic acid (PA) [23]. Then, the dephosphorylation of PA is catalyzed by phospatidate phosphatase to form DAG. A total of seven PA ingredients were identified in this study (Figure 3b and Supplementary Table S3). Interestingly, all the PA ingredients could match the DAG ingredients (Figure 3), which was inconsistent with the previous reports [12,22,24]. The main difference here was that the SIDs had a high relative content of PUFAs in the PA ingredients, such as PA(18:3/18:3), PA(18:2/18:3), and PA(18:2/18:2) (Figure 3b). Nascent FAs from the plastid must be esterified to PC for further desaturation [25,26]. The high content of these PA molecules in developing SIDs implied that those PUFAs desaturated on PC were released into the acyl-CoA pool, where they were assembled into the *sn*-1 and *sn*-2 positions of glycerol-3-phosphate through the Kennedy pathway. Thus, this pathway may play a prominent role in PUFA accumulation in developing SIDs. Also, the results of the PA ingredients further supported the idea of efficient assembly of PUFAs in the *sn*-1 position.

As expected, the 18:2/18:3 and 18:2/18:2 species had a high content both in DAG and PA (Figure 3). An interesting aspect was that the highest content of PA(18:3/18:3) was observed in mature SIDs, but the dephosphorylated production of DAG(18:3/18:3) only ranked sixth in DAG ingredients (Figure 3). A possible explanation for these results could be the substrate selection when using DAG for TAG biosynthesis [23]. Combined with the highest content of TAG(18:3/18:3/18:3) (Figure 2a), we strongly believe that the SID cells may prefer to use DAG(18:3/18:3) as a substrate for the biosynthesis of TAG(18:3/18:3/18:3) during oil accumulation. Extensive use of DAG(18:3/18:3) led to its low ranking in DAG ingredients. Additionally, the highest content of DAG(18:2/18:3) was observed at 110 DAFs (Figure 3a), but the TAG(18:2/18:3/-) category did not occupy a seat in the main TAG ingredients (Figure 2a). This result suggested that the utilization rate of DAG(18:2/18:3) for TAG biosynthesis was low, supporting the conclusion of DAG substrate selection for TAG biosynthesis.

### 2.5. Changes in PC Ingredients during Oil Accumulation

**Figure 4:**
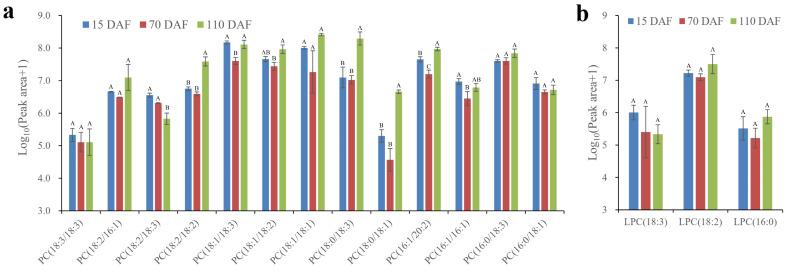
Relative contents of the major (**a**) PC and (**b**) LPC species found in SIDs during development. Please refer to attached Table S4 for details. Values are represented as means ± standard deviations. Different letters above groups represent significant differences, and shared letters represent no significant differences (*p <* 0.01). Abbreviations: phosphatidyl choline, PC; lysophosphatidylcholine, LPC.

In plants, PC clearly functions as an intermediate in TAG biosynthesis, which plays a central role in PUFA assembly [27]. In this study, 13 PC ingredients were identified, mainly including PC(18:1/18:2), PC(18:1/18:3), PC(18:2/18:3), PC(18:0/18:3), and PC(16:0/18:3) (Figure 4a). A previous report indicated that the *sn*-2 acyl group from PC had a crucial effect on PUFA accumulation [28]. Indeed, the ratio of PUFAs at the *sn*-2 position of PC was extremely high in developing SIDs.

To date, three discovered mechanisms involve the release of FA chains from PC to TAG biosynthesis [5]. First, phospholipases catalyze the PC diacylation to form lysophosphatidyl choline (LPC) and acyl-CoA [29]. Second, the FA chains in PC can be interchanged with the FA chains in DAG by PC:DAG cholinephosphotransferase (PDCT) [30]. Third, the FA chains in PC can be used for TAG generation by phospholipid:DAG acyltransferase (PDAT) [22]. As for LPC ingredients, only LPC(18:3), LPC(18:2), and LPC(16:0) were identified (Figure 4b). Apart from the PC(18:3/-), PC(18:2/-), and PC(16:0/-) categories, PC(18:1/18:2), PC(18:1/18:3) and PC(18:0/18:3) contained high PUFA content (Figure 4a). Interestingly, LPC(18:1) and LPC(18:0) were not detected. This finding was unexpected and suggested that different PC categories might be responsible for different mechanisms to provide acyl flux through PC to accumulate PC-modified FAs in TAG. Given the identified LPC ingredients (Figure 4b), we inferred that the PC(18:3/-), PC(18:2/-), and PC(16:0/-) categories, mainly through the first mechanism, release the acyl-CoA for the Kennedy pathway. Conversely, the PC(18:1/-) and PC(18:0/-) categories might be correlated with the generation of PC-derived DAG and TAG through the second and third mechanisms.

## 3. Materials and Methods

### 3.1. Plant Material

The *P. volubilis* specimens are preserved in the Museum of Beijing Forestry University, with the voucher number BJFC 00095003 (https://www.cvh.ac.cn/spms/detail.php?id=9b9d48c8 (accessed on 1 April 2024)). After being introduced from Yunnan, the *P. volubilis* plants were cultivated in the plantation base of Hainan University, Danzhou, Hainan, China (latitude and longitude: 109.503179, 19.542727). This species flowers and fruits year-round, allowing for the continuous collection of samples. Based on our previous investigation of SID development and oil accumulation [14], three crucial time points were selected for lipidome analysis. The three points represent the initiation (15 DAFs), rapid accumulation (70 DAFs), and culmination (110 DAFs) of seed oil accumulation. A short description is provided along with the BBCH code (Supplementary Table S5) [15]. Fruits at stages 731, 787, and 799 were collected from the same tree. At least 25 fruit samples were collected for each period, and fresh seeds were obtained from star-shaped fruits. The samples were immediately flash-frozen in liquid nitrogen and stored at −80 °C until further analysis.

### 3.2. Oil Extraction and FA Analysis

Each sample was a composite formed by mixing together five seeds from the same stage. The mixed sample was freeze-dried using a Yamato DC401/800 freeze dryer (Chongqing, China) until a constant weight was achieved, and samples were then ground into a fine powder. The extraction of SID oil was conducted using Soxhlet extraction as previously reported [31]. FA methyl esters were prepared and detected according to a previous experimental method [22]. Heptadecanoic methyl ester (ANPEL, Shanghai, China) and GLC Mixture (ZZSRM, Shanghai, China) were used as the internal and external standards, respectively. The experiment was repeated three times.

### 3.3. Lipid Extraction

Using the same mixed sample as described above, lipid extraction was performed according to a previously reported method [32]. Briefly, 480 μL MTBE:MeOH (5:1) and 200 μL ddH_2_O were sequentially added to 25 mg of SID samples in an EP tube. After a 30 sec vortex, the samples were homogenized at 35 Hz for 4 min and sonicated for 5 min in an ice-water bath. The homogenization and sonication cycles were repeated 3 times. Then, the samples were incubated at −40 °C for 1 h and centrifuged at 3000 rpm for 15 min at 4 °C. A total of 300 μL of supernatant was transferred to a fresh tube and dried in a vacuum concentrator at 37 °C. Then, the dried samples were reconstituted in 100 μL of 50% methanol in dichloromethane by sonication for 10 min in an ice-water bath. The constitution was then centrifuged at 13,000 rpm for 15 min at 4 °C, and 75 μL of supernatant was transferred to a fresh glass vial for liquid chromatography–tandem mass spectrometry (LC-MS/MS). The quality control sample was prepared by mixing an equal aliquot of the supernatants from all of the samples.

### 3.4. LC-MS/MS Analysis

LC-MS/MS analysis was performed using an ultra-high-performance liquid chromatography system (1290 Infinity II LC System, Agilent Technologies, Santa Clara, CA, USA) equipped with a Kinetex C18 column (2.1 × 100 mm, 1.7 μm, Phenomenex). The mobile phase A consisted of 40% water, 60% acetonitrile, and 10 mmol/L ammonium formate. The mobile phase B consisted of 10% acetonitrile and 90% isopropanol, which was added with 50 mL 10 mmol/L ammonium formate for every 1000 mL mixed solvent. The analysis was carried out with an elution gradient as follows: 0~12.0 min, 40%~100% B; 12.0~13.5 min, 100% B; 13.5~13.7 min, 100%~40% B; and 13.7~18.0 min, 40% B. The column temperature was 55 °C. The auto-sampler temperature was 4 °C, and the injection volume was 2 μL (pos) or 4 μL (neg), respectively.

The QE mass spectrometer was used for its ability to acquire MS/MS spectra in a data-dependent acquisition (DDA) mode in the control of the acquisition software (Xcalibur 4.0.27, Thermo, Waltham, MA, USA). In this mode, the acquisition software continuously evaluates the full scan MS spectrum. The ESI source conditions were set as follows: sheath gas flow rate as 30 Arb, Aux gas flow rate as 10 Arb, capillary temperature as 320 °C (positive) or 300 °C (negative), full MS resolution as 70,000, MS/MS resolution as 17,500, collision energy as 15/30/45 in NCE mode, and spray voltage as 5 kV (positive) or −4.5 kV (negative), respectively.

### 3.5. Data Preprocessing and Statistical Analysis

The raw data files were converted to files in an mzXML format using the ‘msconvert’ program from ProteoWizard. Peak detection was first applied to the MS1 data. The CentWave algorithm in XCMS was used for peak detection with the MS/MS spectrum, and lipid identification was achieved through a spectral match using the LipidBlast library.

SPSS statistical software (version 19) was used for statistical analysis. Differences between groups were determined using a one-way analysis of variance (ANOVA) and multiple-comparison tests. A *p* value less than 0.01 was considered an extremely significant difference.

## 4. Conclusions

The oil content increased from 2.51% to 55.04% with SID maturity. As expected, linoleic and linolenic acid accounted for 78.92% at the maturation period. Using LC-MS/MS analysis, TAG(18:3/18:3/18:3), TAG(18:2/18:2/18:3), TAG(18:1/18:1/18:2), TAG(18:1/18:1/18:1), and TAG(16:0/18:2/18:3) were the main TAG ingredients. The ratio of C18:3/C18:2 in TAG showed a moderate correlation with the corresponding ratio in FA compositions. The ingredients of DAG and PA included 18:2/18:3, 18:2/18:2, 16:0/18:2, and 18:3/18:3, implying efficient accumulation of PUFAs in the *sn*-1 and *sn*-2 positions of TAG. Also, notice that the differences in the contents of the main ingredients among TAG, DAG, and PA might suggest the DAG substrate selection for TAG biosynthesis. The notable differences between LPC and PC ingredients implied that different PC categories might be involved in different mechanisms to provide acyl flux through PC to accumulate PC-modified FAs in TAG. Our lipidomics data from developing SIDs could help to reveal the mechanism of FA assembly during oil accumulation, especially for PUFAs.

## Figures and Tables

**Table 1:** Fatty acid components in developing SIDs.

	Palmitic Acid (C16:0)	Stearic Acid (C18:0)	Oleic Acid (C18:1)	Linoleic Acid (C18:2)	A-Linolenic Acid (C18:3)
15 DAFs	36.84 ± 2.92% ^A^	22.47 ± 1.70% ^A^	6.02 ± 0.37% ^C^	20.20 ± 1.11% ^B^	14.47 ± 0.60% ^B^
70 DAFs	8.14 ± 0.71% ^B^	6.02 ± 0.28% ^B^	12.36 ± 0.54% ^A^	30.80 ± 1.21% ^A^	42.68 ± 1.25% ^A^
110 DAFs	5.34 ± 0.38% ^B^	4.76 ± 0.58% ^B^	10.98 ± 0.31% ^B^	33.84 ± 0.73% ^A^	45.08 ± 0.66% ^A^

Note: Different letters represent significant differences, and shared letters represent no significant differences (*p <* 0.01).

## Data Availability

All data generated or analyzed during this study are included in this published article.

## Supplementary Materials

The following supporting information can be downloaded at https://www.mdpi.com/article/10.3390/plants13162193/s1. Table S1: LC-MS/MS data for TAG species; Table S2: LC-MS/MS data for DAG species; Table S3: LC-MS/MS data for PA species; Table S4: LC-MS/MS data for PC and LPC species; Table S5: Description of the phenological stages of *P. volubilis* fruits by the BBCH code.
